# Antimicrobial Prophylaxis for the Prevention of Surgical Site Infections in Orthopaedic Oncology - A Narrative Review of Current Concepts

**DOI:** 10.7150/jbji.39050

**Published:** 2019-10-15

**Authors:** Daniel Müller, Dominik Kaiser, Kati Sairanen, Thorsten Studhalter, İlker Uçkay

**Affiliations:** 1 Department of Orthopedic Surgery, Balgrist University Hospital, Zurich, Switzerland; 2 Unit of Clinical and Applied Research, Balgrist University Hospital, Zurich, Switzerland; 3 Infectious Diseases and Infection Control, Balgrist University Hospital, Zurich, Switzerland

**Keywords:** orthopaedic surgery, oncology, antibiotic prophylaxis, prevention, review

## Abstract

The incidence of surgical site infections (SSIs) after elective tumour orthopaedic surgery in adults is higher than non-oncologic orthopaedic surgery. Their causative microorganisms and antibiotic susceptibilities are also different from the non-oncologic cases; with no apparent predictable microbiological patterns. Clinicians continue to struggle to tailor the optimal prophylactic regimen for the very heterogeneous group of tumour patients. Many clinicians thus prolong the first-and second-generation cephalosporin agents, while a minority chooses to broaden the antimicrobial spectrum by combination prophylaxis. The variability in current practices and surgical techniques is enormous, even within the same setting. The scientific literature lacks adequate retrospective case-studies and there is currently only one prospective randomized trial. In this narrative review, we discuss various perioperative antibiotic concepts in oncologic orthopaedic surgery, including a summary of the state-to-the-art, opinions and difficulties related to the different prophylactic strategies.

## Introduction

In elective orthopedic oncologic surgery (musculoskeletal malignancies), surgical site infections (SSI; 1) occur much more frequently than after non-oncologic surgeries. The incidence of oncologic SSI has been reported from 4 to 38% 2, largely depending on the case-mix, but being certainly higher than for any other elective orthopedic surgery. SSI rates in non-oncologic orthopedic surgery usually range from 0.1% to 4% 1. Postoperative wound complications in orthopedic tumor surgery are equally high, ranging from 16% to 56% 3-5.

The reasons for these higher SSI incidences are presumably multifactorial for the majority of episodes: extensive soft tissue dissections and large remaining cavities 3, long operating times 6-8, mega-implants 9, prior radiotherapy 6,10, immune-suppression 6 with oncologic treatments 11,12 and iterative surgeries (e.g. second resections or plastic coverage of large defects; 3). A local immune-suppression, beyond the systemic one, is possible. The irradiated skin might equally enhance the SSI risk 3. Besides additional costs, oncologic SSIs are serious complications that may have a relevant influence on the patient's survival time 13. Without a doubt, they certainly prolong the length of the stay in acute hospital settings 4,14 and may postpone a mandatory postoperative chemotherapy.

While the perioperative antibiotic prophylaxis is well established for the vast majority of orthopedic interventions, and endorsed by international 15 or national 16 guidelines, clinicians still struggle to tailor an optimal prophylactic regimen for elective orthopedic tumor surgery. Clinicians use different antibiotic regimens and this difference is huge between the centers and author groups 17. Antibiotic overuse is the logical consequence, since prolongation of the antibiotic prophylaxis is common despite the lack of a proven effect 1,18,19, comparable to non-oncologic orthopedic surgery, not even for open fractures 18. In contrast, such a prolongation can lead to a skin colonization of multi-resistant bacteria 19,20. From a theoretical stand point, there are multiple concepts that are discussed: combination of local and systemic antimicrobial agents, an enlargement of the antibiotic spectrum or a prolonged prophylaxis with either standard or enhanced antibiotic regimens, or not to change the standard antibiotic prophylaxis at all. Because the prophylaxis effect is only a minor part of the overall prevention procedure, and cannot influence the inherently high incidence of (multi-resistant) SSIs. The antibiotic prophylaxis itself may reduce the SSI burden by 3-8%, but rarely by more 1 and targets the usual microbiology. *Staphylococcus aureus* and β-hemolytic streptococci are the hallmarks of orthopedic SSIs, while coagulase-negative staphylococci and other skin commensals are witnessed in implant-related infections. Gram-negative pathogens, including anaerobes, are seldom in orthopedic surgery 1, but may predominate in traumatology settings, especially open fractures 18.

In this narrative review, we highlight various perioperative prophylactic concepts in elective orthopedic tumor surgery, including a summary of the presumed state-to-the-art, opinions and difficulties. We cover 1) the epidemiology of these SSI's, the skin colonization of multi-resistant germs in these oncologic patients, 2) the differences according to the anatomical localizations, 3) the microbiology and the theoretical minimal antibiotic coverage necessary to prevent SSIs as best as possible, 4) the major prophylaxis strategies would theoretically yield the greater benefit in terms of prevention: enlarging the spectrum or prolonging the standard prophylaxis? In contrast, we do not address infection treatment 8, surgical techniques, or non-antibiotic preventions of SSIs 3,10,21, for which a broader literature is available 3,11.

## Methods

All authors performed a scientific search for literature on PubMed and Google (Scholar) for publications regarding SSIs among adult oncologic orthopedic patients using the MeSH terms “cancer”, “oncology”, “orthopedic”, “surgery”, “sarcoma”, “bone”, “soft tissue” together with “infection” and “prophylaxis” in English, Turkish, Finnish, French and German languages until 30 June 2019. We also specifically scoured available (orthopedic) SSI prevention guidelines. Even if we first detected the abstracts, we always read the full article. According to international use, we defined SSI as a bacterial infection at the former uninfected surgical operation site occurring up to 30 days after the non-implant-related index surgery, or within one year if an implant became infected. In a next step we hand-searched references of retrieved papers for further articles and excluded pediatric reports, cases with community-acquired infections, repeat publications, animal studies, *in vitro* experiments, open (pathologic) fracture surgery in the cancer site 18, hematogenous SSIs 22, mixed orthopedic and other (visceral) surgeries, surgery for diagnostic purposes only, surgical cure by simple distant amputation, cryosurgery, and the prevention of various nosocomial infections other than SSI 23 in orthopedic oncologic patients.

Community-acquired infections were the opposite of nosocomial (healthcare-associated) infections, whereas hematogenous infections were due to secondary seeding to the surgical site via a bacteremic spread originating from a remote infection site. We also excluded reports of cosmetic and/or benign lipoma surgeries, because we estimated them distinct from classical (sarcoma) surgery in terms of clinical entities and surgical techniques. Our search strategy was similar to the PARITY review 24, with however new publications since 2012 and a broadening of the literature search for non-English languages.

For the sake of practicability, we renounced on differentiating between elective and traumatic oncologic surgery upon pathological fractures, between superficial and deep SSIs 4,14, between primary cancer surgery and metastases, or the reasons of SSIs (“direct” SSI or indirectly by contaminated allografts during the same first intervention); or due to second looks during the same hospitalization. The reasons for not distinguishing superficial and deep SSIs were multiple: a) most oncologic orthopedic articles do not differentiate between deep organ and superficial scar infections, b) many orthopedic cancers have superficial and deep parts, c) our goal was not to compare the numbers between the centers, and d) this distinction is interesting for epidemiological surveillances and therapies, but not regarding antibiotic prophylaxis. In the entire literature of SSI prevention 1,15, scientific recommendations do not stratify between deep and superficial infections in terms of antibiotic prophylaxis. Likewise, we ignored the nature of the postsurgical immune-suppressive therapy, the use of vacuum-assisted negative pressure therapy or plastic surgical interventions after initial orthopaedic surgery, which also may influence overall SSI risk. The minimal clinical follow-up time in the individual papers was left to the discretion of the author groups; without any prerequisite from our side.

## Results

**Figure 1:**
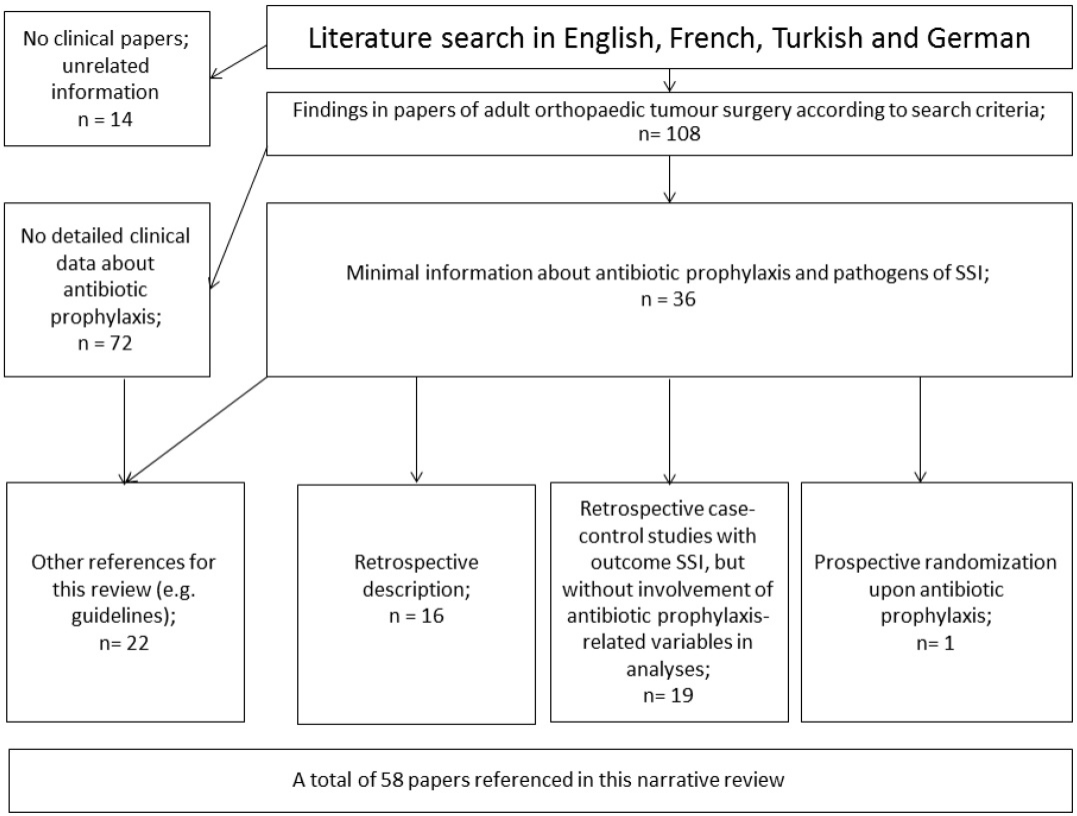
Flowchart of the literature search

By 30 June 2019, we retrieved 108 articles, which are sixty more since the extensive and English-language literature review of the PARITY author group (*discussed later on*) in 2012 24. However, 72 reported no information regarding the antibiotic prophylaxis or pathogens which was a minimal requirement to be discussed in this narrative review. Figure 1 displays the flowchart of the article selections and the final results. Almost all articles were written by surgeons (sometimes in non-surgical journals) and were retrospective, with one exception 25. None was primarily written by infectious diseases physicians, or professionals with experience in infection control. In contrast, infectious diseases physicians and surgeons wrote the guidelines and official recommendations together. Strikingly, the USA, Japan, Italy and Korea (*in descending order*) reveal the most publications, with methicillin-resistant *S. aureus* (MRSA) being a frequent causative oncologic SSI pathogen in Japan compared to other countries. The majority of the publications stem from the last two decades.

We finally resumed 20 relevant publications in Table 1 and analyzed 22 others for our review. This literature was very heterogeneous regarding different settings, implants, antibiotic-loaded bone cements, anatomical localizations, reported co-morbidities, methods of diagnosing infection, different details of information, methodology, outcomes of interest, skin incisions, tumor volumes, antimicrobial prophylaxes, pathogens, amputation versus limb-sparing surgery, surgical techniques and follow-up times 26. A systemic statistical evaluation, let alone a (pseudo)-meta-analysis regarding the estimated impact of different prophylaxes were therefore deemed impossible. Importantly, even if many papers retrospectively performed multivariate analyses targeting the outcome “occurrence of SSI”, the majority of them forgot to include any prophylactic-related variable in their final models; even though the perioperative antibiotic prophylaxis is a cornerstone in SSI prevention 1,15,27. Moreover, even if prophylaxis was mentioned, almost no publication mentions the adequacy in terms of its timing 1. This again illustrates the general rule composition of the medical disciplines among the authors in this particular field of publication, sparing anesthesiologists or internists such as infectious diseases physicians.

### Microbiology of oncologic orthopedic SSI (know your enemy)

Even though it is a major subject of debate and a prerequisite to tailor specific prophylaxes, there is an astonishing paucity of microbiologic data regarding SSIs in oncologic orthopedic patients, and their various subsets of surgeries and conditions. The causative microorganisms of oncologic orthopedic SSIs seem very different from non-oncologic orthopedic surgery 25 and more resistant towards the standard recommended prophylactic regimens such as with first- or second-generation cephalosporins, clindamycin or glycopeptides 1 (Table 1). SSI outbreaks among oncologic orthopedic patients have not (yet) been described.

The very scarce data advocate that this microbiology is highly individual 25 with no apparent predictable microbiological patterns. One author of this review recently investigated the nature of these SSI in a single-center retrospective study 25, by excluding skin colonization and subsequent episodes of infections. Among 2752 different first episodes of various orthopedic infections, only 14 (0.5%) concerned SSI at the site of prior oncologic surgery. Then the authors added a literature review and found, all together with their own data, that oncologic orthopedic patients reveal no more prior antibiotic therapy (before intraoperative sampling) than non-oncologic patients, but witness more enterococci 11,25,35, Gram-negatives 11,17,25,35 (of which one third can be non-fermenting rods that are naturally resistant to standard prophylaxis 17, polymicrobial infections 11,17,25,35, or infections due to multi-resistant skin commensals 11,25,35. In contrast, the proportion of classic SSI pathogens such as *S. aureus* or streptococci were not different from the control group 17,25. Of note, although this was the largest comparative microbiological analysis in oncologic orthopedic SSIs, it must be clear that no major conclusions can be drawn from only 14 cases 25.

### Anatomical localization of tumor surgery

The anatomical localization of tumor surgery may play a role. The SSI risk is strikingly higher in the lower extremities, the pelvis and the spine 14 (10-15%), whereas SSIs (1-5%)^11^,^35^,^37^,^38^ are rarely witnessed in the upper extremity or the hand. SSIs in pelvic surgery reach incidences of more than 20% (7,31,32, Table 1).

### Skin colonization of resistant pathogens among orthopedic cancer patients

Specifically regarding the orthopedic cancer population and although a prerequisite to tailor specific prophylaxes, we could not find any published reports linking pre-surgical colonization with multi-resistant microorganisms such as extended-spectrum beta-lactamase-producing (ESBL) rods 39 or MRSA 40, at any body localization, with the occurrence of orthopedic SSI with these pathogens.

### Duration of antibiotic prophylaxis

Various first-or second generation cephalosporins were the most frequently used prophylactic agents, but their prophylactic durations varied between 1 and 5 days (17, Table 1). Many authors maintain a standard prophylactic preoperative antibiotic regimen of cephalosporin for a maximum of 24 hours after surgery despite reporting SSI rates of up to 15% 10,28 and do not discuss their attitude and motives 14,28. A large Japanese center with almost 500 own cases remains with a 24 h prophylaxis, even though many of their cases involve technically difficult surgeries such as hip disarticulations, pelvectomies and others 2. Another report from a highly specialized Italian research group advocates that a single-shot of cefazolin 2 g would be enough 19. Ziranu et al. divided their oncologic orthopedic adult patients into two risk classes (low risk with single-shot cefazolin 2g IV 30 minutes before surgery and high risk with three doses of cefazolin 2g IV) and found no difference in terms of SSI (3 SSIs/33 procedures vs. 8/49 procedures; 19). Single-shot prophylaxes with cefuroxime remain also valid in many other centers 25. Pushing the limits, Saddegh et al. resected 103 soft tissue sarcomas. Despite the lack of peri-operative prophylaxis in half of their patients, the SSI risk was similar (21% SSI incidence with prophylactic antibiotics versus 26% without; 4).

If there is modification of the standard perioperative antibiotic prophylaxis, the majority of the research groups prolong it without changing the agents (Table 1). This means not to refer to a larger antibiotic spectrum, but rather a prolonged administration of the standard antibiotic. Examples are Hettwer et al. achieving 3.6% SSI incidence after tumor hip arthroplasty by individually prolonging the standard parenteral cefuroxime 1.5g tid to an average of 7.4 days (range, 2-28 days) 29 or Piccioli et al. administering cefazolin prophylaxis until the removal of the wound drainage 34. Importantly, all author groups prolonging the prophylaxis do not indicate why they really do so.

### Antibiotic-loaded bone cements and local prophylaxes

Interestingly, the orthopaedic oncologic literature does not provide much insight into antibiotic-loaded bone cements during arthroplasty surgery of mega-prostheses 1, which is difficult to obtain. Although antibiotic-loaded bone cement is of interest in all cemented arthroplasties, a very large number of patients are needed when studying the effect of antibiotic loaded bone cements and no conclusions can be drawn from small studies of mega-prostheses. Regarding tumor surgery, one center reports that they renounce on local antibiotic prophylaxis 17, whereas another acknowledges the systematic use of local gentamicin 33. Other groups choose a midway and apply gentamicin-containing bone cements in selected patients (roughly 25%) 7. Of note in this latter study, the use of gentamicin-loaded bone cement (despite a presumed susceptibility of the later SSI pathogen) did not reduce SSIs when compared to without local gentamicin (7; *p*=0.9).

### Enlarging the antibiotic spectrum

We found no comparative studies questioning on the enlargement of the prophylactic antibiotic spectrum. Such a broadening could empirically target methicillin-resistant Gram-positive pathogens, obligate anaerobes or non-fermenting Gram-negative rods; solely or all together. A retrospective case-control reported a 7% SSI risk in spinal metastases surgery by a cefepime prophylaxis of an unknown duration 6. Hardes et al., also retrospectively, reported a prophylaxis composed of local gentamicin and third-generation parenteral cephalosporins during 3-7 days; followed by an oral second-generation cephalosporin until total wound healing 33. Their SSI risk was still 18% despite the use of at least three different agents. Peel et al. unilaterally broadened the Gram-positive spectrum by using vancomycin in 47% of their tumor surgeries. However, compared to the control group with standard cephalosporin prophylaxis, the vancomycin use did not reduce the SSI risk 8. Angelini et al. used the same approach among 270 pelvic resections, but this time combining cefazolin and intravenous tobramycin (a practically-total gram-negative coverage) during five days. Their SSI incidence of 20% was not inferior to other research groups 31. Hasan et al., from the PARITY trial 24 group, performed a survey 41. Of the 72 (Canadian) oncologic orthopaedic surgeons responding to the questionnaire, the respondents varied considerably in their choices of regimens and dosages. Although 73% prescribe a first-generation cephalosporin, 25% favor additional coverage with an aminoglycoside and/or vancomycin. Furthermore, only a third of them believed that antibiotics could be stopped after 24 hours latest, but 41% continued with their prophylaxis until the drains were removed 41.

### Specific antimicrobial prophylaxes without antibiotic agents

This group mainly consists of silver-coated mega-prostheses in lower extremity cancer surgery, in addition to usual perioperative systemic antibiotic prophylaxis. The difficulty is to determine if silver-coating would be superior to standard systemic antibiotic prophylaxes, in as much as the coating is frequently not applied at the articulating surfaces 33. Several author groups advocate the benefit of silver 28, which we frequently encounter in other domains of (implant-related) infectious diseases. Silver has garnered much interest because of its excellent antimicrobial activity coupled with low toxicity 34. Silver-coating for tumor endoprostheses was also the subject of another published narrative literature review 35. Its authors, together with displaying a 10% SSI risk among their own cases, advocate an overall SSI risk from 2.2 to 11.8% despite silver-coating 35. Another research group, with a fixed cefazolin-based systemic prophylaxis, compared 38 silver-coated hip mega-prostheses to 30 uncoated titan hip mega-prostheses after an average follow-up of four years 28. The corresponding SSI incidences were 7.9% (3 cases) versus 16.7% (5 cases) in favor of the silver-coating, but this difference was in-significant (Fisher exact-test: *p*=0.45) and the study was underpowered. Regarding late infections, the difference was non-existing even in crude group comparison 28. Another specialized research group lacked early SSIs among silver-coated mega-prostheses compared to two-third of early SSIs in uncoated arthroplasties, and a SSI risk of 12% versus 23% in favor of the silver-coating. There was again no statistical difference when examining the subgroup of late SSI (after 6 months following the index arthroplasty) between both prosthetic groups (12% versus 8%) 34. A third group compared 51 sarcoma patients with silver-coated hip arthroplasties to 74 similar titan implants 33. The crude SSI incidences were 6% and 18%, respectively (two-sided Fisher-exact-test; *p*=0.06). However, the authors noticed significantly higher revision surgeries due to mechanical failures in the titan prostheses group 33. On the electron microscope, the researchers saw a disruption of the prosthetic surface in some explanted prostheses with few, small silver grains present on the surface, whereas this visualized silver was completely absent on other prostheses 34. A local or systemic toxicity of silver-coating, per analogy to possible metal debris in arthroplasties, does not seem to play a clinical role 28,34. In summary, the studies of silver-coating are mostly underpowered, but seem to reveal a benefit in terms of less SSIs on the short term (during the first couple of weeks), but not after several months 28,34.

### Guidelines and expert recommendations

There are many local 32, regional, national (Switzerland, Scotland, USA 16,42), administration (CDC; 43), or international recommendations (WHO; 15) regarding SSI prevention in (orthopaedic) surgery. Most of them are regularly updated and their last versions stem from 2017 to 2019 19. When reading these guidelines, we failed to find any particular prophylactic regimens for orthopaedic oncologic surgery. In Switzerland, the country of this narrative review, the latest *SwissNoso* recommendations, issued by a panel of experts in infection control, do not provide recommendations for cancer surgery in general, let alone specifically for oncologic orthopaedic surgery 44. The same applies to the German recommendations 45.

### Retrospective studies

There are many retrospective comparative studies assessing the postsurgical SSI risk in oncologic orthopaedic patients, but most fail to introduce antibiotic prophylaxis into their univariate or multivariate models 6,8,9,12,14,21,33,34,36,46-49. Their variable of interests is rather the surgical approach, the patient's race, steroid use, blood loss, nutrition, irradiation, or the serum albumin levels. Even a study analyzing more than 800 orthopaedic cancer surgeries renounced on introducing prophylactic antibiotic variables into its final multivariable models 9. Similarly, due to the small number of oncologic patients, many general orthopaedic SSI risk assessments do not stratify among oncologic patients among their study populations 50,51.

### Prospective studies

We only found the announcement of one prospective-randomized trial regarding perioperative antibiotic prophylaxis in adult lower-extremity endoprosthetic tumour surgery. It is named the PARITY trial (*Prophylactic Antibiotic Regimens in Tumour Surgery; ClinicalTrials.gov NCT01479283*), comparing two prophylactic cefazolin durations (1 vs. 5 days). This is a blinded, randomized controlled trial, using a parallel two-arm design. It stems from Canada, is multicentric with 37 sites in seven countries, and has one year of active follow-up for each participating patient 24,52. The study started in 2012. Randomization is blocked, with block sizes known only to the methods center responsible for randomization, and stratified by location of tumor and study center. Following their pilot evaluation, the investigators target a study population of 600-1200 patients (alpha 0.05, power 80%, estimated 50% reduction of the relative risk). According to *ClinicalTrials.gov,* it still continues with already 600 episodes included, and is scheduled until December 2020. Unless there is protocol modification, patients 15 years of age or older who are undergoing surgical excision and endoprosthetic reconstruction of a primary bone tumor will receive either short (24 h) or long (5 days) duration postoperative antibiotics. Exclusion criteria include prior surgery or infection within the planned operative field, known colonization with methicillin-resistant *S. aureus* or vancomycin-resistant enterococci at enrolment. The primary outcome will be the risk for deep SSIs in each arm. Secondary outcomes will include type and frequency of antibiotic-related adverse events, patient functional outcomes and quality-of-life scores, reoperation and mortality 24.

## Discussion

Among adult orthopaedic patients undergoing elective oncologic surgery, an English-language literature search by the PARITY study group in 2012 identified 48 eligible studies resuming a weighted SSI mean of 9.5% (95% confidence interval 8.1% to 11.0%; 24). Their review could not analyze the impact of the perioperative antibiotic prophylaxis, probably also because of the fact that studies issuing from the same hospital are likely to use the same prophylaxis for all patients. We added twenty more publications in 2019 (Table 1) and equally failed to estimate the individual impacts of various prophylactic regimens. The sparse literature was too heterogeneous. Even a (pseudo)-meta-analysis or an evaluation of the antibiotic prophylaxis in a multivariate analysis were impossible.

Nevertheless, we can resume some facts, which seem to be well documented. First, the anatomical localization is of great importance. Orthopaedic oncology surgery of the upper extremity has similar SSI rates than non-oncologic orthopaedic interventions 11,35,37,38, reducing the need to tailor a specific antibiotic prophylaxis for this group. The problem group are patients with surgery on the lower extremity, the lumbar spine and the pelvic region, as oncologic SSI rates are markedly higher in these localizations 11,35,37,38. Further investigations on specific antibiotic prophylaxis in this patient collective may yield benefits.

We ignore the exact reasons for this discrepancy between upper and lower body sites. It might be that the lower extremity tumours harbour a bigger size, that surgery in weight-bearing extremities is more conservative (in contrast to easier amputations in the arms), that the clinical detection of lower-extremity cancers is more delayed compared to the thinner arms, that the curative surgery in the spine and pelvis is more cumbersome, longer and deeper when compared to the arm, or that the pelvis and calves are in proximity of the genital and intestinal regions, making local Gram-negative skin colonization more likely.

As patients with upper extremity cancers remain mobile and are often managed as outpatients before surgery (in compared to orthopaedic spine and pelvic cancer patients who cannot always walk), it could also be that the latter group yields a significantly longer pre-surgical stay with increasing theoretical risk of (nosocomial) multi-resistant skin colonization. These remain speculations. Many research groups investigated the influence of a delay between admission and surgery with the occurrence of subsequent SSI and its pathogen profile. These studies mostly concerned open fractures 18,51 and do not address the usual delay times in orthopaedic oncology. A Genevian study investigated the pre-surgical hospitalization time in a multivariate model for implant-related non-oncologic orthopaedic surgery, and found no association of longer hospital stays with more antibiotic-resistant SSIs 51.

A second finding of our review is that the SSIs of adult oncologic orthopedic patients reveal substantially more enterococci, Gram-negative pathogens (including *Pseudomonas aeruginosa* and anaerobes), or multi-resistant skin commensals compared to non-oncologic SSIs 19,20,25. Strikingly, we encountered the discrepancy between “classical” non-oncologic SSI microorganisms with a predominance of *S. aureus*, streptococci (and skin commensals in implant-related orthopedic surgery) in every article; independently of the setting, the underlying cancer, preexisting antibiotic therapy, prior local radiotherapy or the anatomical localization (Table 1). As no outbreak situation *sensu strictu* 1 has been described in all these retrieved publications, we presume that this discrepancy is genuine, for which we ignore the exact reasons.

These resistant pathogens are not covered by standard orthopedic antibiotic prophylaxis comprising first and second generation cephalosporins (or vancomycin and clindamycin in case of beta-lactam allergy; 1). We thus question the utility of standard antibiotic prophylaxis, which might not be enough. What are alternative regimens? Basing on theoretical considerations, we think there are five options (plus eventually: antimicrobial-coating of arthroplasties): a) prolonging the standard prophylaxis beyond the single-shot; b) enlarging the antibiotic spectrum; or c) a combination of a) and b), or avoiding to administer new prophylactic agents in addition to the current therapeutic antimicrobial treatment. This would be a similar situation as in high-grade open fractures, another field of orthopedic and trauma surgery that has been neglected by scientific research for a long time. In high-grade open fractures, neither the prolongation of antibiotic prophylaxis beyond three days 18, nor the enlargement of the antibiotic spectrum to glycopeptides or carbapenems has led to a reduction of the overall infection risk situated in between 10-30% 53 so far. As in orthopedic cancer surgery, in open fractures direct comparative studies randomizing cephalosporins to prophylactic carbapenems are lacking. This also applies to the prevention of infections in ischemic areas of an amputation stump 54,55.

Concerning general orthopedic surgery, and especially prosthetic surgery, the literature is full of opinion papers and retrospective studies investigating the possibility of better outcomes with broader prophylaxis 51. The propositions differ from one paper to another and focus on different strategies which are: continuing the prophylaxis beyond a single dose, increase of dose, combining with local prophylaxis (e.g. topical vancomycin powder in spine surgery), double prophylaxis against Gram-negative, Gram-positive, methicillin-resistant strains and anaerobes, or by investigating the performance of universal glycopeptid prophylaxis. In summary, the majority of these enhancements have failed to reduce SSI risk in orthopedic surgery 1,18,51. Exceptions remain rare, very specific and often not reproducible by other research groups 51. Furthermore, an enlarged antibiotic prophylaxis may potentially even be harmful 51, especially with prophylactic aminoglycosides against Gram-negative pathogens. Numerous studies reported transient kidney injuries by amino-glycosides or combined vancomycin prophylaxis in orthopedic surgery. The risk for antibiotic-resistant organisms seems to be negligible 51. Local antimicrobial technologies might help. However, we lack large epidemiological data that these procedures would be superior in preventing SSI when compared to systemic antibiotic administration.

It is unclear how much the orthopedic surgical world is aware of the microbiologic discrepancy between oncologic SSIs, standard prophylaxis and possible solutions. During the first 2013 Philadelphia Consensus Meeting on Periprosthetic Joint Infections, several hundred delegates (orthopedic surgeons, infectious diseases physicians, microbiologists and others) debated about unresolved issues in the field of osteo-articular infections. One question was: “*What is the recommended prophylaxis, in patients undergoing major orthopedic reconstructions for either tumor or non-neoplastic conditions using mega-prosthesis*?” The expert audience acknowledged the lack of scientific data and 93% of them voted that “*Until the emergence of further evidence, we recommend the use of routine antibiotic prophylaxis for patients undergoing major reconstruction*.” 56. This prolongation of standard perioperative cephalosporin prophylaxis might be a solution, at least partially. So far, the international community is betting on this solution. The aforementioned PARITY trial compares two prophylactic cefazolin durations (1 vs. 5 days; 41). This trial is of utmost importance for the scientific community, but may not be enough, as it only investigates a subgroup (but nevertheless important group) of lower-extremity endoprosthetic tumour surgery 24. More importantly, according to our retrospective assessment of the microbiology of the oncologic orthopedic SSI, cefazolin or cefuroxime would fail to cover the majority of the infecting pathogens we have noted, independently of their duration of prophylaxis. Based on the enemy we regularly detect, we believe that the enlargement of the antimicrobial spectrum towards all methicillin-resistant pathogens and Gram-negatives could be more beneficiary. This needs to be proven in a prospective randomized (multicenter) study; like the ongoing PARITY trial 24, only with other antimicrobial agents and a standardized shorter duration; i.e. 24h.

At last, but comprising an entirely hypothetical option is the concept of an individualized antibiotic prophylaxis. Such a prophylaxis would target known skin colonizers and/or the individual microbiome. Already in non-oncologic orthopedic surgery a minority of pathogens regularly may escape to standard antibiotic prophylaxis. This usual resistance ranges from roughly six 57 to 49% in oncologic surgery patients 58 or 70 percent especially among orthopedic *S. epidermidis* implant infections 59. Theoretically, this individual prophylaxis could avoid the risk of nonspecific enlargement of coverage which would expose the medical environment to greater antibiotic pressure and generation of more resistant organisms. There may be also local epidemiological variations in the susceptibility pattern that have to be considered. However, these solutions remain purely theoretical, since the microbiome evaluations has started only recently and the concept of an individualized prophylaxis has not yet been proven to the best of our knowledge 1,15.

## Conclusion and Outlook

In this narrative review, we discuss various perioperative antibiotic concepts in oncologic orthopedic surgery. Most SSI literature in adult elective orthopedic cancer surgery is written by surgeons who operate on these complex patients. Accordingly, the focus of their publications can be different from the major interests of infectious diseases physicians who read them. Many questions of high importance remain open in terms of perioperative prophylaxis, even if the PARITY trial will soon come to its end.

The overall SSI risk among orthopedic oncologic surgeries is high; ranging from 10-15% while most literature (and consequently also the authors of this review did not differentiate between deep and pure superficial SSIs 1. The risk of SSI is markedly higher in the pelvic region and the lower extremity compared to the upper extremity, while the spine region has an intermediate risk. Maybe oncologic orthopedic patient care should include different prophylactic regimens based on the localization of their tumor. The microbiology of orthopedic SSI in adult oncologic patients is significantly different than for non-oncologic elective surgery. More targeted (and prospective) studies are needed in terms of choice of the anti-microbial agents, rather than the duration of the standard prophylaxis. Hopefully, these future publications will involve more infectious disease physicians and/or other specialists in infection control. A prospective-randomized trial comparing a standard cephalosporin prophylaxis to a broad antibiotic spectrum, but without necessary prolonging the duration of application, would be very welcome. According to our personal opinion and as a theoretical example, a possible prophylactic antibiotic coverage for lower extremity, pelvic and spine tumor surgery could include a combination of single-dose vancomycin (against Gram-positive pathogens) and single-dose gentamicin (large Gram-negative coverage), while a formal coverage of anaerobic pathogens would be less important 25,60. The role of silver-coating implants could be further evaluated equally in non-arthroplasty implants. The skin carriage of resistant pathogens among adult orthopedic cancer patients needs further research. Likewise, we equally need more studies targeted to identity organisms (e.g. with 16S deep sequencing).

## Figures and Tables

**Table 1:** Prophylaxis of surgical site infections (SSI) in orthopaedic cancer surgery (selected literature)

Author	Setting	No. cases	No. of SSI (%)	Prophylactic agent (s)	Duration prophylaxis	SSI *S. aureus*	SSI *Gram -negative rods*	SSI *enterococci*	SSI *polymicrobial*	SSI skin* germs
				*Short prophylaxis -Cephalosporin*						
Ziranu (19)	All cancers	93	11 (12%)	Cefazolin 2 g	Single-shot; 24h	n.r.	n.r.	n.r.	n.r.	n.r.
Nagano (2)	All cancers	457	19 (4%)	“standard”	24 h	10 (53%)	4 (22%)	0 (0%)	n.r.	4 (22%)
Sebaaly (14)	Spine	297	15 (5%)	Cefazolin 2 g	24 h	11 (73%)	n.r.	n.r.	n.r.	n.r.
Donati (28)	Mega-prostheses	68	8 (12%)	Cefazolin 2 g	24 h	n.r.	n.r.	n.r.	n.r.	n.r.
Sugita (10)	Spine	279	41 (15%)	Cefazolin 2 g	24 h	n.r.	n.r.	n.r.	n.r.	n.r.
Rod-Fleury (25)	All cancers	n.r.	14	Cefuroxime	4-24 h	3 (21%)	10 (71%)	6 (43%)	10 (71%)	7 (50%)
					*Long prophylaxis - cephalosporins*					
Hettwer (29)	Prostheses	111	4 (4%)	Cefuroxime	2-28 days	n.r.	n.r.	n.r.	n.r.	n.r.
Morii (30)	Soft tissue	84	7 (8%)	n.r.	at least 72 h	4 (57%)	2 (29%)	0 (0%)	1 (14%)	2 (29%)
Angelini (31)	Pelvis	270	55 (20%)	Cefazolin, tobramycin	5 days	n.r.	n.r.	n.r.	n.r.	n.r.
Sanders (17)	Periacetabular reconstructions	70	18 (26%)	cephalosporin	1-5 days	2 (11%)	16 (89%)	5 (28%)	14 (78%)	6 (33%)
					*Long broad-spectrum prophylaxis*					
Rossi (32)	All cancers	723	63 (9%)	cephalosporinvancomycin, amino-glycos	2-5 days	27 (47%)	24 (38%)	16 (10%)	29 (46%)	10 (16%)
Hardes (33)	Mega-prostheses	125	16 (13%)	Silver, local gentamicin, cephalosporin	10-20 days	1 (6%)	0 (0%)	3 (19%)	4 (25%)	8 (50%)
Peel (8)	Prostheses	121	17 (14%)	Vancomycin, cephalosporin	0-22 days	5 (29%)	3 (18%)	2 (12%)	3 (18%)	6 (35%)
Saddegh (4)	Soft tissue	103	16 (16%)	n.r.	0-some days	8 (50%)	3 (19%)	0 (0%)	n.r.	3 (19%)
Piccioli (34)	Limbs	30	5 (17%)	Cefazolin 2 g	48-72h	n.r.	n.r.	n.r.	n.r.	n.r.
Ozaki (7)	Pelvis	22	8 (36%)	Standard + genta cement	3-4 months	5 (23%)	7 (32%)	2 (9%)	n.r.	0 (0%)
					*Unprecise duration*					
Demura (6)	Spine	97	7 (7%)	Cefepime	n.r.	2 (29%)	2 (29%)	1 (14%)	1 (14%)	3 (43%)
Schmolders (35)	Mega-prostheses	100	10 (10%)	Standard plus silver-coating	n.r.	0 (0%)	6 (60%)	5 (50%)	5 (50%)	4 (40%)
Lee (36)	Bone cancer	316	31 (10%)	n.r.	n.r.	7 (23%)	3 (10%)	0 (0%)	n.r.	5 (16%)
*Resumee*	*All cancer*	*3506*	*365 (10%)*	*various*	*24h or more*	*33%*	*40%*	*18%*	*46%*	*26%*

n.r. = not reported; * = coagulase-negative staphylococci, corynebacteria, cutibacteria. Of note, the proportions of different pathogen categories may exceed 100%, as many infections may reveal different pathogens within the same episode.

## References

[1] Uçkay I, Hoffmeyer P, Lew D, Pittet D (2013). Prevention of surgical site infections in orthopaedic surgery and bone trauma: state-of-the-art update. J Hosp Infect.

[2] Nagano S, Yokouchi M, Setoguchi T (2014). Analysis of surgical site infection after musculoskeletal tumor surgery: risk assessment using a new scoring system. Sarcoma.

[3] Geller DS, Hornicek FJ, Mankin HJ, Raskin KA (2007). Soft tissue sarcoma resection volume associated with wound-healing complications. Clin Orthop Relat Res.

[4] Saddegh MK, Bauer HC (1993). Wound complication in surgery of soft tissue sarcoma. Analysis of 103 consecutive patients managed without adjuvant therapy. Clin Orthop Relat Res.

[5] Sanniec KJ, Swanson S, Casey WJ 3rd, Schwartz A, Bryant L, Rebecca AM (2013). Predictive factors of wound complications after sarcoma resection requiring plastic surgeon involvement. Ann Plast Surg.

[6] Demura S, Kawahara N, Murakami H (2009). Surgical site infection in spinal metastasis: risk factors and countermeasures. Spine (Phila Pa 1976).

[7] Ozaki T, Hillmann A, Bettin D, Wuisman P, Winkelmann W (1996). High complication rates with pelvic allografts. Experience of 22 sarcoma resections. Acta Orthop Scand.

[8] Peel T, May D, Buising K, Thursky K, Slavin M, Choong P (2014). Infective complications following tumour endoprosthesis surgery for bone and soft tissue tumours. Eur J Surg Oncol.

[9] Miwa S, Shirai T, Yamamoto N (2019). Risk factors for surgical site infection after malignant bone tumor resection and reconstruction. BMC Cancer.

[10] Sugita S, Hozumi T, Yamakawa K, Goto T, Kondo T (2016). Risk factors for surgical site infection after posterior fixation surgery and intraoperative radiotherapy for spinal metastases. Eur Spine J.

[11] Mavrogenis AF, Papagelopoulos PJ, Coll-Mesa L, Pala E, Guerra G, Ruggieri P (2011). Infected tumor prostheses. Orthopedics.

[12] McPhee IB, Williams RP, Swanson CE (1998). Factors influencing wound healing after surgery for metastatic disease of the spine. Spine (Phila Pa 1976).

[13] Atkinson RA, Davies B, Jones A, van Popta D, Ousey K, Stephenson J (2016). Survival of patients undergoing surgery for metastatic spinal tumours and the impact of surgical site infection. J Hosp Infect.

[14] Sebaaly A, Shedid D, Boubez G (2018). Surgical site infection in spinal metastasis: incidence and risk factors. Spine J.

[15] Organization WH (2016). Global Guidelines for the Prevention of Surgical Site Infection. World Health Organization.

[16] SIGN publication no.104 Antibiotic prophylaxis in surgery. 2014. http://www.sign.ac.uk.

[17] Sanders PTJ, Bus MPA, Scheper H (2019). Multiflora and Gram-Negative Microorganisms Predominate in Infections Affecting Pelvic Endoprostheses Following Tumor Resection. J Bone Joint Surg Am.

[18] Dunkel N, Pittet D, Tovmirzaeva L (2013). Short duration of antibiotic prophylaxis in open fractures does not enhance risk of subsequent infection. Bone Joint J.

[19] Ziranu A, Lillo M, Fantoni M, Maffulli N, Maccauro G (2018). Single dose cefazolin is safe and effective for pre-operative prophylaxis in orthopaedic oncology. J Biol Regul Homeost Agents.

[20] Harbarth S, Samore MH, Lichtenberg D, Carmeli Y (2000). Prolonged antibiotic prophylaxis after cardiovascular surgery and its effect on surgical site infections and antimicrobial resistance. Circulation.

[21] Gradl G, de Witte PB, Evans BT, Hornicek F, Raskin K, Ring D (2014). Surgical site infection in orthopaedic oncology. J Bone Joint Surg Am.

[22] Uçkay I, Lübbeke A, Emonet S (2009). Low incidence of haematogenous seeding to total hip and knee prostheses in patients with remote infections. J Infect.

[23] Sax H, Uçkay I, Balmelli C (2011). Overall burden of healthcare-associated infections among surgical patients. Results of a national study. Ann Surg.

[24] Ghert M, Deheshi B, Holt G (2012). Prophylactic antibiotic regimens in tumour surgery (PARITY): protocol for a multicentre randomised controlled study. BMJ Open.

[25] Rod-Fleury T, Uçkay I (2019). Microbiological Particularities of Surgical Site Infections in Oncologic Orthopedic Surgery Compared to Non-Oncologic Surgery-Single Center Experience and Literature Review. Clin Surg.

[26] Tsuchiya H, Wan SL, Sakayama K, Yamamoto N, Nishida H, Tomita K (2005). Reconstruction using an autograft containing tumour treated by liquid nitrogen. J Bone Joint Surg Br.

[27] Surgeons AAoO (2014). Recommendations for the Use of Intravenous Antibiotic Prophylaxis in Primary Total Joint Arthroplasty. Public Relations Department. American Academy of Orthopaedic Surgeons. Information Statement.

[28] Donati F, Di Giacomo G, D'Adamio S (2016). Silver-Coated Hip Megaprosthesis in Oncological Limb Savage Surgery. Biomed Res Int.

[29] Hettwer WH, Horstmann PF, Hovgaard TB, Grum-Scwensen TA, Petersen MM (2015). Low infection rate after tumor hip arthroplasty for metastatic bone disease in a cohort treated with extended antibiotic prophylaxis. Adv Orthop.

[30] Morii T, Mochizuki K, Tajima T, Ichimura S, Satomi K (2012). Surgical site infection in malignant soft tissue tumors. J Orthop Sci.

[31] Angelini A, Drago G, Trovarelli G, Calabro T, Ruggieri P (2014). Infection after surgical resection for pelvic bone tumors: an analysis of 270 patients from one institution. Clin Orthop Relat Res.

[32] Rossi B ZC, Toma L, Ferraresi V, Biagini R (2016). Surgical site infections in treatment of musculoskeletal tumors: Experience from a single oncologic institution. J Orthop Oncol.

[33] Hardes J, von Eiff C, Streitbuerger A (2010). Reduction of periprosthetic infection with silver-coated megaprostheses in patients with bone sarcoma. J Surg Oncol.

[34] Piccioli A, Donati F, Giacomo GD (2016). Infective complications in tumour endoprostheses implanted after pathological fracture of the limbs. Injury.

[35] Schmolders J, Koob S, Schepers P (2017). Lower limb reconstruction in tumor patients using modular silver-coated megaprostheses with regard to perimegaprosthetic joint infection: a case series, including 100 patients and review of the literature. Arch Orthop Trauma Surg.

[36] Lee JA, Kim MS, Kim DH (2009). Postoperative infection and survival in osteosarcoma patients. Ann Surg Oncol.

[37] Wittig JC, Simpson BM, Bickels J, Kellar-Graney KL, Malawer MM (2001). Giant cell tumor of the hand: superior results with curettage, cryosurgery, and cementation. J Hand Surg Am.

[38] Wittig JC, Bickels J, Kellar-Graney KL, Kim FH, Malawer MM (2002). Osteosarcoma of the proximal humerus: long-term results with limb-sparing surgery.

[39] Agostinho A, Renzi G, Haustein T (2013). Epidemiology and acquisition of extended-spectrum beta-lactamase-producing Enterobacteriaceae in a septic orthopedic ward. SpringerPlus.

[40] Uçkay I, Lübbeke A, Harbarth S (2012). Low risk despite high endemicity of methicillin-resistant Staphylococcus aureus infections following elective total joint arthroplasty: a 12-year experience. Ann Med.

[41] Hasan K, Racano A, Deheshi B (2012). Prophylactic antibiotic regimens in tumor surgery (PARITY) survey. BMC Musculoskelet Disord.

[42] Surgeons AAoO (2014). Recommendations for the Use of Intravenous Antibiotic Prophylaxis in Primary Total Joint Arthroplasty.

[43] Berrios-Torres SI, Umscheid CA, Bratzler DW (2017). Centers for Disease Control and Prevention Guideline for the Prevention of Surgical Site Infection, 2017. JAMA Surg.

[44] Senn L VD, Widmer A (2015). Zanetti G, Kuster S. Aktualisierte Empfehlungen zur perioperativen Antibiotikaprophylaxe in der Schweiz, 2015. SwissNoso.

[45] Prävention postoperativer Wundinfektionen (2018). Empfehlungen der Kommission für Krankenhaushygiene und Infektionsprävention (KRINKO) beim Robert-Koch-Institut. Bundesgesundheitsbl.

[46] Severyns M, Briand S, Waast D, Touchais S, Hamel A, Gouin F (2017). Postoperative infections after limb-sparing surgery for primary bone tumors of the pelvis: Incidence, characterization and functional impact. Surg Oncol.

[47] Puchner SE, Funovics PT, Bohler C (2017). Oncological and surgical outcome after treatment of pelvic sarcomas. PLoS One.

[48] Pascal-Moussellard H, Broc G, Pointillart V, Simeon F, Vital JM, Senegas J (1998). Complications of vertebral metastasis surgery. Eur Spine J.

[49] Kumar S, van Popta D, Rodrigues-Pinto R (2015). Risk factors for wound infection in surgery for spinal metastasis. Eur Spine J.

[50] Berbari EF, Hanssen AD, Duffy MC (1998). Risk factors for prosthetic joint infection: case-control study. Clin Infect Dis.

[51] Davat M, Wuarin L, Stafylakis D (2018). Should antibiotic prophylaxis before orthopedic implant surgery depend on the duration of pre-surgical hospital stay?. Antimicrob Resist Infect Control.

[52] Rendon JS, Swinton M, Bernthal N (2017). Barriers and facilitators experienced in collaborative prospective research in orthopaedic oncology: A qualitative study. Bone Joint Res.

[53] Gonzalez A, Suva D, Dunkel N (2014). Are there clinical variables determining antibiotic prophylaxis-susceptible versus resistant infection in open fractures?. Int Orthop.

[54] Dunkel N, Belaieff W, Assal M (2012). Wound dehiscence and stump infection after lower limb amputation: risk factors and association with antibiotic use. J Orthop Sci.

[55] Jeys LM, Grimer RJ, Carter SR, Tillman RM (2005). Periprosthetic infection in patients treated for an orthopaedic oncological condition. J Bone Joint Surg Am.

[56] Gehrke T, Parvizi J (2013). 2013 Proceedings of the International Consensus Meeting on Periprosthetic Joint Infection.

[57] Misteli H, Widmer AF, Rosenthal R (2011). Spectrum of pathogens in surgical site infections at a Swiss university hospital. Swiss Med Wkly.

[58] Teillant A, Gandra S, Barter D (2015). Potential burden of antibiotic resistance on surgery and cancer chemotherapy antibiotic prophylaxis in the USA: a literature review and modelling study. Lancet Infect Dis.

[59] Uçkay I, Harbarth S, Ferry T (2011). Methicillin-resistance in orthopaedic coagulase-negative staphylococcal infections. J Hosp Infect.

[60] Lebowitz D, Kressmann B, Gjoni S (2017). Clinical features of anaerobic orthopaedic infections. Infect Dis (Lond).

